# Pharmacokinetics and Safety of Vortioxetine in Pediatric Patients

**DOI:** 10.1089/cap.2016.0155

**Published:** 2017-08-01

**Authors:** Robert L. Findling, Adelaide S. Robb, Melissa DelBello, Michael Huss, Nora McNamara, Elias Sarkis, Russell Scheffer, Lis H. Poulsen, Grace Chen, Ole Michael Lemming, Johan Areberg, Philippe Auby

**Affiliations:** ^1^ Department of Psychiatry and Behavioral Sciences, Kennedy Krieger Institute and Johns Hopkins University, Baltimore, Maryland.; ^2^ Department of Psychology and Behavioral Health, Children's National Health Systems, Washington, District of Columbia.; ^3^ Department of Psychiatry and Behavioral Neuroscience, Cincinnati Children's Hospital Medical Center, Cincinnati, Ohio.; ^4^ Universitätsmedizin, Klinik für Kinder- und Jugendpsychiatrie und -Psychotherapie, Mainz, Germany.; ^5^ University Hospitals Case Medical Center, Cleveland, Ohio.; ^6^ Sarkis Family Psychiatry, Gainesville, Florida.; ^7^ Department of Psychiatry and Behavioral Sciences, University of Kansas School of Medicine, Wichita, Kansas.; ^8^ H. Lundbeck A/S, Valby, Denmark.; ^9^ Clinical Pharmacology, Takeda Development Center Americas, Deerfield, Illinois.

**Keywords:** vortioxetine, pharmacokinetics, children, adolescents, dosing, adverse events, antidepressant

## Abstract

***Objective:*** The primary objectives of this study were to evaluate the pharmacokinetics (PK) and tolerability of single and multiple doses of vortioxetine in children and adolescents with a depressive or anxiety disorder and to provide supportive information for appropriate dosing regimens for pediatric clinical trials.

***Methods:*** This prospective, open-label, multinational, multisite, multiple-dose trial enrolled 48 patients (children and adolescents; 1:1 ratio) divided into 8 cohorts (4 adolescent and 4 child), with each cohort including 6 patients. The cohorts in each age group were assigned to receive one of four dosing regimens: vortioxetine 5, 10, 15, or 20 mg q.d. for 14 days. The total treatment period lasted 14–20 days with patients in the higher dose cohorts uptitrated over 2–6 days. Plasma samples for PK analysis were obtained on the first and last days of dosing.

***Results:*** Among children and adolescents, respectively, 62% and 92% had depression and 58% and 33% had anxiety disorder. Comorbid attention-deficit/hyperactivity disorder (ADHD) was present in 50% of children and 38% of adolescents. After 14 days q.d. at the target dose, the PK of vortioxetine concentrations was generally proportional to the dose in both age groups. Exposure, as assessed by maximum plasma concentrations and area under the plasma concentration–time curve from time 0 to 24 hours, was 30%–40% lower in adolescents than in children. There was no significant relationship between sex, height, or ADHD diagnosis and PK parameters. Most adverse events were mild in severity and consistent with those seen in adults.

***Conclusion:*** The results suggest that the dosages of vortioxetine evaluated (5–20 mg q.d.; approved for treatment in adults) and the uptitration schedule used are appropriate for pediatric efficacy and safety trials.

## Introduction

Mood disorders are among the most debilitating illnesses in children and adolescents. Depression and anxiety are directly associated with a substantial burden among this age group worldwide, including an increased risk of recurrent episodes and an increased risk of suicide (National Institute for Health and Care Excellence 2005; Kessler 2007; Birmaher et al. 2007; Bourgeois et al. 2012; Avenevoli et al. 2015). However, to date, only two antidepressants (both selective serotonin reuptake inhibitors) are approved for use in the pediatric population for the treatment of depression: fluoxetine is approved for major depressive disorder (MDD) in the United States and the European Union for children and adolescents (ages ≥8 years) and escitalopram for MDD in the United States for adolescents only (ages 12–17 years).

Vortioxetine is a multimodal antidepressant that acts as a 5-HT_3_, 5-HT_7_, and 5-HT_1D_ receptor antagonist, 5-HT_1B_ receptor partial agonist, 5-HT_1A_ receptor agonist, and inhibitor of the 5-HT transporter *in vitro* (Bang-Andersen et al. 2011; Mork et al. 2012; Westrich et al. 2012). The pharmacokinetics (PK) of vortioxetine in adults is characterized by prolonged absorption, an oral clearance of 33 L/h, a large volume of distribution, and an average elimination half-life of 66 hours (Areberg et al. 2014). Metabolism of vortioxetine occurs primarily through oxidation through multiple cytochrome P450 (*CYP450*) isozymes (predominantly *CYP2D6*), with subsequent glucuronic acid conjugation (Hvenegaard et al. 2012). The major carboxylic acid metabolite is pharmacologically inactive, and *in vitro* data suggest that vortioxetine and its metabolites are unlikely to inhibit or induce a large number of *CYP450* enzymes (Food and Drug Administration Center for Drug Evaluation and Research 2012; European Medicines Agency 2013; Chen et al. 2015).

Vortioxetine is approved in the United States and the European Union for the treatment of MDD and major depressive episodes, respectively, in adults at a dose range of 5–20 mg q.d. Vortioxetine has also demonstrated efficacy in the treatment of adults with generalized anxiety disorder in some (Bidzan et al. 2012) but not in all trials (Rothschild et al. 2012; Mahableshwarkar et al. 2014).

There are numerous challenges in the clinical development of treatments for pediatric patients with depression or anxiety, with many negative or failed trials. Several factors likely contributed to these failures, including inappropriate dosing regimens (Findling et al. 2006). Given the historical lack of PK and dose-finding studies in children and adolescents, the usual method of body weight adjustment of the dose may result in inappropriate doses, with either subtherapeutic doses that result in a failure to detect a positive efficacy signal or a too high dose unnecessarily putting children and adolescents under a higher safety risk (Moreno et al. 2007).

A recent meta-analysis identified a higher rate of serious adverse events for available antidepressants in children as opposed to adults, possibly indicating an inappropriate dosing regimen as a result of a lack of phase II studies (i.e., dose-ranging studies) in pediatric patients (Sharma et al. 2016). Therefore, identifying evidence-based dosing strategies remains a key initial step in drug development for pediatric use. PK and dose-finding studies can provide important information about how best to dose medications in children and adolescents (Findling et al. 2006).

To respond to regulatory requests in the United States (Food and Drug Administration 2011) and the European Union (European Medicines Agency 2006) and to address issues regarding optimization of study designs evaluating vortioxetine in children and adolescents, this first international pediatric PK study with vortioxetine was designed to determine whether the dose range approved for adult patients is appropriate for use in pediatric efficacy and safety studies (ClinicalTrials.gov identifier: NCT01491035; EudraCT No.: 2010-020170-42). The primary objective was to describe the PK and tolerability of single and multiple doses of vortioxetine in children and adolescents with a diagnosis of depressive or anxiety disorder. The secondary objective was to evaluate the safety of vortioxetine over the dose range evaluated. Ultimately, these data would guide future dosing strategies for pediatric safety and efficacy studies.

## Methods

### Patients

Pediatric outpatients of ages 7–17 years with a *Diagnostic and Statistical Manual of Mental Disorders*, Fourth Edition, Text Revision (DSM-IV-TR™) diagnosis of depressive or anxiety disorder at screening that warranted antidepressant therapy (as judged by the investigator) were eligible for inclusion. Females of childbearing potential were required to have a negative pregnancy test at screening and to use adequate contraception throughout the study and for 30 days poststudy completion.

As attention-deficit/hyperactivity disorder (ADHD) is commonly diagnosed in children with depression and/or anxiety, comorbid ADHD was allowed and concomitant stable treatment with a stimulant (i.e., minimum of 4-week stable period before study treatment) was accepted at sites in the United States but not in Germany.

Participants were excluded if they had a history of an Axis I diagnosis of bipolar disorder, posttraumatic stress disorder, autism, pervasive developmental disorder, obsessive-compulsive disorder, schizophrenia, or schizoaffective disorder; were unable to maintain a stable dose of ADHD medication for ≥4 weeks before study treatment; and had a current diagnosis or history of substance abuse or alcohol abuse <6 months before screening or testing positive at screening for drugs of abuse.

Patients also were not eligible if they were at significant risk of suicide; had clinically significant abnormal vital signs or electrocardiogram (ECG); tested positive for hepatitis B surface antigen or hepatitis C virus antibody; had abnormal tests suggesting renal, liver, or thyroid disease; had a disease or took medication that could, in the investigator's opinion, interfere with the assessment of safety, tolerability, or efficacy, or interfere with the conduct or interpretation of the study; or had any general medical condition or were taking a concomitant medication that might affect the PK of vortioxetine. Patients also were not allowed to have participated in a clinical study within 30 days of screening.

### Study design

This was a prospective, multinational, multisite, open-label, multiple-dose trial that was conducted by seven principal investigators at six sites in the United States and one site in Germany. The study consisted of a 2-week screening/washout period, a 14–20-day treatment period, and a safety follow-up visit 14 days after the last dose of vortioxetine (only for patients not continuing into the extension period).

The study was designed and conducted in accordance with the principles of the *Declaration of Helsinki*, and the protocol was approved by the Rhineland-Palatine State Medical Association (Germany) and the following U.S. institutional review boards: University Hospitals Cleveland Medical Center Institutional Review Board for Human Investigation, Western Institutional Review Board, Johns Hopkins Medicine Institutional Review Board, and University of Kansas School of Medicine Human Subjects Committee. All patients provided assent to participate and the parent(s)/legal representative(s) provided written informed consent before the study.

The study consisted of eight cohorts (four adolescent and four child cohorts), each including six patients. The cohorts were sequential within each age group with patients assigned to receive vortioxetine 5, 10, 15, or 20 mg once daily (q.d.) for 14 days. Patients were instructed to take study medication at the same time each day, preferably in the morning.

**FIG. 1.**
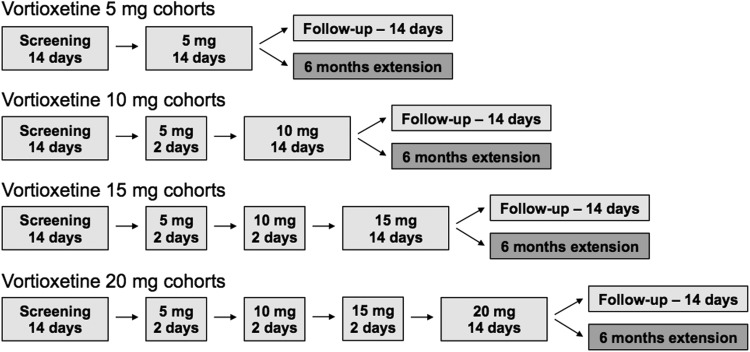
Study design overview.

All patients received vortioxetine 5 mg on the first day of dosing and the total treatment period lasted 14–20 days with vortioxetine uptitrated for 2, 4, or 6 days in patients assigned to the 10, 15, and 20 mg dose groups, respectively (Fig. 1). The adolescent 5 mg q.d. cohort and then the adolescent 10 mg q.d. cohort were initiated first. After that, the child 5 mg q.d., adolescent 15 mg q.d., child 10 mg q.d., adolescent 20 mg q.d., child 15 mg q.d., and child 20 mg q.d. cohorts were started sequentially. In this way, adolescent patients were exposed to a specific dose of vortioxetine before the child patients received the same dose. The preliminary safety, tolerability, and PK data from each cohort were evaluated by an external data safety monitoring board before the progression of the next sequential cohort.

Patients preferably were asked to remain at the investigational site from the safety baseline (day −1) until the last blood sample had been collected on day 2 and during the last 2 days (i.e., morning of the last treatment day until all study-related assessments were completed on the following morning). However, patients were allowed to leave the site if they were considered clinically stable in the opinion of the investigator and if the investigator confirmed acceptable tolerability of vortioxetine. Treatment adherence was assessed by the use of a study-provided diary. In addition, patients were asked to return all unused study medication. Patients who completed the main study period, if judged advisable by the investigator, were offered to continue in an optional, 6-month, open-label, flexible-dose extension trial (Findling et al. 2016).

### Bioanalysis

Blood samples for PK parameter estimates were collected before the first dose of vortioxetine on day 1 and at 1, 3, 5, 8, 12, and 24 hours after the first dose. In addition, on the last treatment day (days 14, 16, 18, or 20) after the patient's final dose of vortioxetine, blood samples were collected at the same time points as on day 1. Samples were analyzed using protein precipitation followed by ultraperformance liquid chromatography with tandem mass spectrometric detection (Kall et al. 2015), a method validated in accordance with the EMA *Guidance on Bioanalytical Method Validation* (European Medicines Agency 2006) and the FDA *Guidance for Industry* (Food and Drug Administration 2001). The lower limit of quantification for vortioxetine was 0.20 ng/mL, with a linear range of 0.20–100 ng/mL.

### PK analysis

All patients who took ≥1 dose of vortioxetine who had sufficient postdose sampling data for estimation of PK parameters were included in the PK analysis. The PK of vortioxetine was evaluated by means of nonlinear mixed effect analysis (population PK) using the software NONMEM^®^ version 7.3 NONMEM (ICON Development Solutions, Ellicott City, MD). A two-compartment model—parameterized in terms of absorption rate constant (ka), oral clearance (CL/F), central volume of distribution (V2/F), intercompartmental clearance (Q/F), peripheral volume of distribution (V3/F), and lag-time (ALAG)—was used as previously developed for analyzing the PK of vortioxetine in adults (Areberg et al. 2014).

The impact of the covariates age, sex, body size, ethnicity, study site, ADHD diagnosis, and coadministration with a stimulant on PK parameters was evaluated by adding the covariate–parameter relationship with the model and comparing the result with the base model (i.e., without the relationship). The covariate–parameter relationships were tested in a forward inclusion/backward exclusion manner to avoid influence from correlated covariates. Based on the individual parameter values from the nonlinear mixed effect analysis (*post hoc* estimates), the following derived parameters were estimated:

• t_½_: elimination half-life
• AUC_0–24_: area under the plasma concentration–time curve from zero to 24 hours postdose

Maximum plasma concentration (C_max_) and time to C_max_ (t_max_) were obtained directly from the observed data, both for day 1 and for the last day of dosing in the main study period (days 14, 16, 18, or 20).

### CYP2D6 genotyping

Genotyping of patients for *CYP2D6* was performed for explorative interpretation of the PK results (i.e., not statistically tested) as vortioxetine is mainly metabolized by *CYP2D6* based on *in vitro* and *in vivo* data and as *CYP2D6* polymorphism may impact the PK of vortioxetine in pediatric patients. Blood samples (2.4 mL) for genotyping were analyzed for the following alleles to infer metabolic status: CYP2D6 *3, *4, *5, *6, *9, *10, *17, *29, *41, and gene duplication (*1xN, *2xN, etc.).

### Safety and tolerability

Safety and tolerability assessments included adverse events, laboratory tests (hematology, clinical chemistry, and urinalysis), vital signs (blood pressure, heart rate, respiratory rate, and body temperature), weight, ECG, physical examination, the Columbia Suicide Severity Rating Scale (C-SSRS), and the Pediatric Adverse Event Rating Scale (PAERS). The PAERS utilized in this study is a clinician-rated scale consisting of 45 items (43 specific signs and symptoms and 2 to be specified) and is designed to assess adverse events occurring in pediatric patients treated with psychotropic medication in clinical studies (Shapiro et al. 2009). Adverse events and vital signs were assessed at baseline (day −1), on days 1, 2, 4, 6, and 13 of treatment, at the end of treatment, and at the safety follow-up (adverse events only). Laboratory tests and ECGs were performed at baseline and at the end of treatment.

For recording adverse events at each visit, patients and their caregivers who accompanied them to study visits were asked a nonleading question (i.e., “How do you feel?”). Adverse events that were spontaneously reported by the patient and/or their caregivers or were observed by the investigator were recorded and assessed by the investigator for severity and relationship with study medication. Adverse events were classified by the investigator as mild (i.e., causes minimal discomfort and does not interfere in a significant manner with the patient's normal activities), moderate (i.e., is sufficiently uncomfortable to produce some impairment of the patient's normal activities), or severe (i.e., is incapacitating and preventing the patient from participating in the patient's normal activities). Adverse events were coded by appropriately qualified personnel using the lowest level term according to the Medical Dictionary for Regulatory Activities (MedDRA), Version 16.1. The PAERS was completed after these open nonleading questions.

### Statistics

The PK, safety, and tolerability data were summarized using descriptive statistics.

## Results

### Patient distribution and demographics

Of the 48 patients enrolled, 47 completed the study and 41 (19 children, 22 adolescents) continued onto the 6-month extension period. One girl in the adolescent 5 mg q.d. cohort was lost to follow-up and withdrawn from the study because of nonadherence of visits (i.e., did not complete the second PK visit). Therefore, the PK analysis included 48 patients on the first day of dosing and 47 patients on the last day. Six patients chose not to go into the extension period for reasons that included lack of efficacy (*n* = 2), too much of a commitment (*n* = 1), patient going away for college (*n* = 1), missing parental consent (*n* = 1), and no stated reason (*n* = 1).

Baseline characteristics and demographics are summarized in Table 1. A concurrent diagnosis of ADHD was present in 12 (50%) children and 9 (38%) adolescents. Of these, 11 patients (8 children, 3 adolescents) were treated with stimulants for their ADHD during the main study period. Adherence, as assessed by patient diary and tablet count, was high, with the majority of patients reported receiving all doses of study medication. The overall mean adherence was 98%.

**Table 1.** Patient Demographics and Baseline Characteristics

	*Child cohort*					*Adolescent cohort*				
	*5 mg (*n = *6)*	*10 mg (*n = *6)*	*15 mg (*n = *6)*	*20 mg (*n = *6)*	*Total (*n = *24)*	*5 mg (*n = *6)*	*10 mg (*n = *6)*	*15 mg (*n = *6)*	*20 mg (*n = *6)*	*Total (*n = *24)*
Sex, n (%)										
Male	3 (50)	3 (50)	4 (67)	4 (67)	14 (58)	3 (50)	3 (50)	1 (17)	2 (33)	9 (38)
Female	3 (50)	3 (50)	2 (33)	2 (33)	10 (42)	3 (50)	3 (50)	5 (83)	4 (67)	15 (63)
Mean age, years (SD)	10.3 (1.2)	9.7 (1.2)	10.5 (0.8)	9.8 (1.8)	10.1 (1.3)	15.7 (1.9)	15.3 (1.9)	15.2 (1.2)	14.8 (1.9)	15.3 (1.6)
Race, *n* (%)										
White	4 (67)	2 (33)	3 (50)	4 (67)	13 (54)	4 (67)	4 (67)	4 (67)	4 (67)	16 (67)
Black/AA	1 (17)	4 (67)	3 (50)	2 (33)	10 (42)	1 (17)	2 (33)	1 (17)	2 (33)	6 (25)
Other	1 (17)	0	0	0	1 (4)	1 (17)	0	1 (17)	0	2 (8)
Mean weight, kg (SD)	52.0 (25.1)	41.1 (15.8)	44.9 (5.7)	40.1 (19.3)	44.5 (17.4)	74.2 (13.5)	79.1 (19.9)	71.6 (38.8)	54.8 (15.9)	69.9 (24.4)
Mean height, cm (SD)	145.2 (11.9)	143.3 (14.2)	148.9 (7.5)	137.3 (11.5)	143.7 (11.6)	170.2 (7.9)	166.1 (9.6)	162.6 (10.8)	161.8 (9.3)	165.2 (9.5)
Mean BMI, kg/m^2^ (SD)	23.7 (7.7)	19.3 (4.0)	20.3 (3.1)	20.7 (6.7)	21.0 (5.6)	25.7 (4.8)	28.4 (5.6)	26.4 (11.7)	21.0 (6.2)	25.4 (7.6)
Depressive disorder, *n* (%)					15 (62)					22 (92)
Anxiety disorder, *n* (%)					14 (58)					8 (33)
Depressive disorder + anxiety disorder, *n* (%)					5 (21)					6 (25)

Some percentages do not add to 100% because of rounding.

AA, African American; BMI, body mass index; SD, standard deviation.

### Single-dose PK

**FIG. 2.**
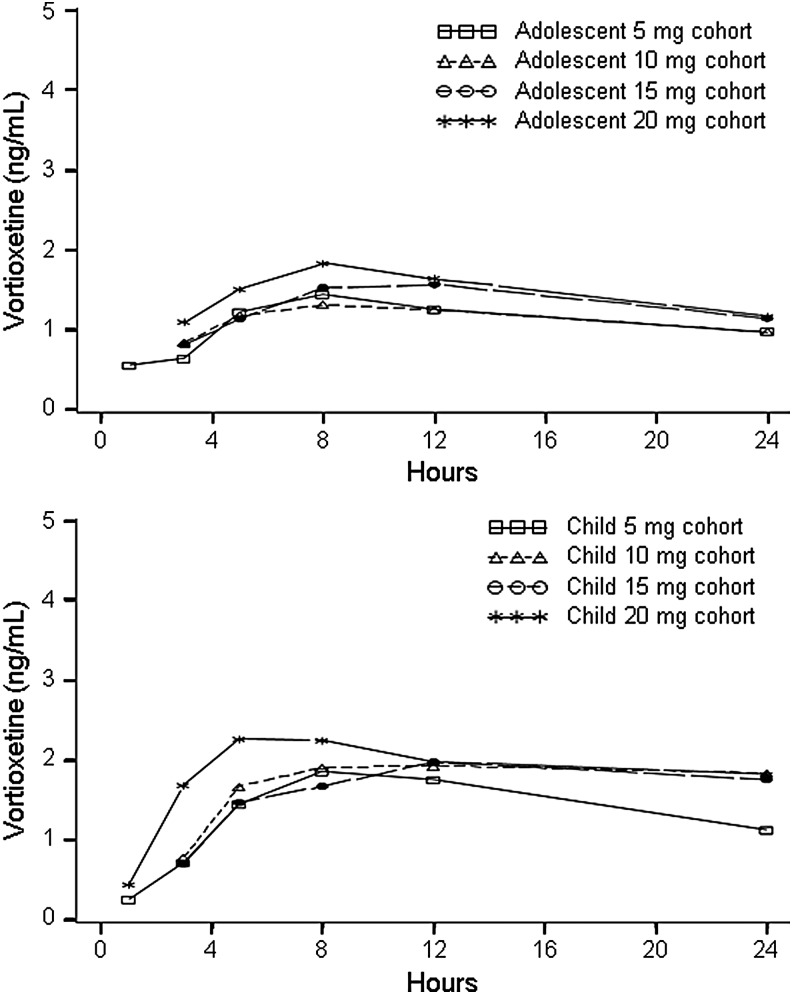
Mean plasma concentrations of vortioxetine in adolescents (upper) and children (lower) on the first day of dosing (i.e., all patients received vortioxetine 5 mg).

On the first day of dosing, all patients received vortioxetine 5 mg. The plasma concentration profile of vortioxetine in children and adolescents on the first day is illustrated in Figure 2. Among the children, the mean ± standard deviation C_max_ was 2.2 ± 0.7 ng/mL, with the median t_max_ occurring 8.0 hours (range, 4.6–23.8 hours) after the dose. The mean AUC_0–24_ among all 24 children was 38.7 ± 11.0 ng/(h·mL) after the first dose. Among adolescents, mean C_max_ was 1.6 ± 0.6 ng/mL, median t_max_ was 8.1 hours (range, 2.9–24.0 hours), and mean AUC_0–24_ was 27.9 ± 8.0 ng/(h·mL) after the first dose. In general, exposures (as assessed by C_max_ and AUC_0–24_) were lower in adolescents than in children. The median C_max_ ratio between children and adolescents was 1.40 and the corresponding ratio for AUC_0–24_ was 1.34.

### Multiple-dose PK

**FIG. 3.**
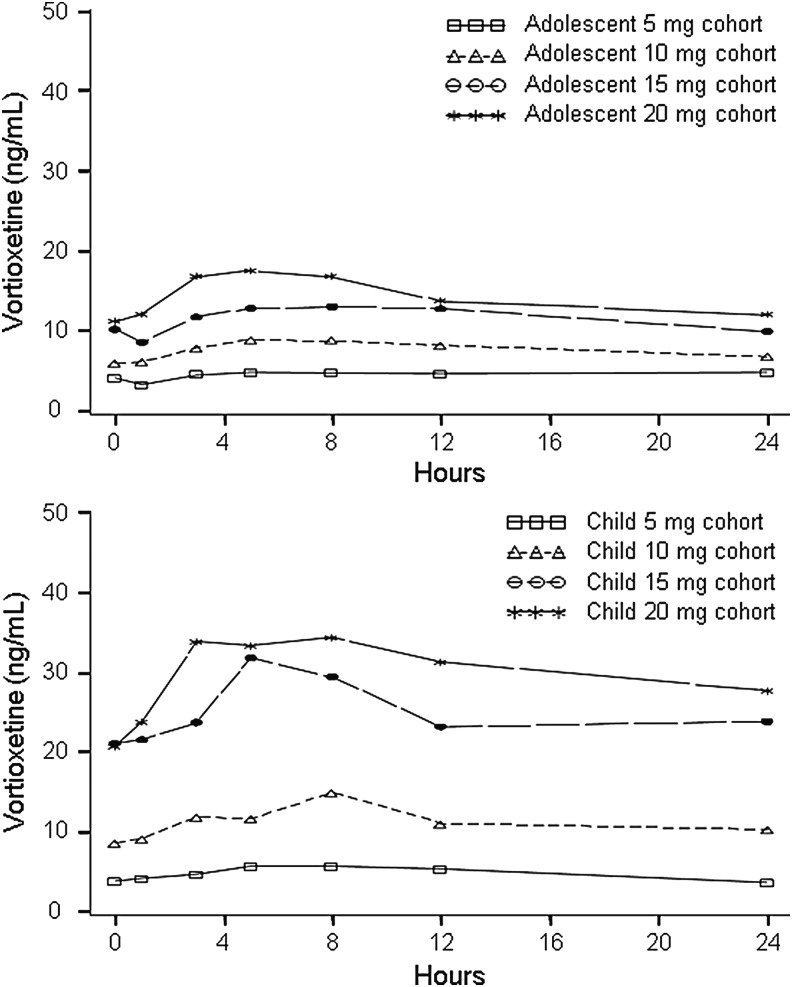
Mean plasma concentrations of vortioxetine in adolescents (upper) and children (lower) on the last day of dosing.

The mean plasma concentration–time profile for vortioxetine on the last day of dosing after 14 days of treatment at the target dose is illustrated in Figure 3. The profile suggests that plasma concentrations are approximately proportional to dose in both children and adolescents. Median PK parameters after the last dose are summarized in Table 2. Similar to the single-dose PK, exposures were lower in adolescents than in children on the last day of dosing: the median ratio between dose-normalized C_max_ for children and adolescents was 1.54, and the corresponding ratio for AUC_0–24_ was 1.55. The median CL/F of vortioxetine was lower in children versus adolescents (38 L/h vs. 59 L/h, respectively) and the median t_1/2_ was longer (60 hours vs. 47 hours, respectively).

**Table 2.** Pharmacokinetic Parameters of Vortioxetine After the Last Dose (Day 14, 16, 18, or 20)

	*Child cohort*					*Adolescent cohort*				
*Median (SD)*	*5 mg (*n = *6)*	*10 mg (*n = *6)*	*15 mg (*n = *6)*	*20 mg (*n = *6)*	*Total (*n = *24)*	*5 mg (*n = *5)*	*10 mg (*n = *6)*	*15 mg (*n = *6)*	*20 mg (*n = *6)*	*Total (*n = *23)*
C_max_, ng/mL^a^	5.0 (3.3)	14 (8.2)	26 (21)	31 (20)	–	4.3 (3.7)	7.8 (2.8)	15 (6.2)	16 (8.1)	—
t_max_, hours (range)^a^	5.0 (4.7–7.9)	6.4 (4.9–8.0)	8.0 (5.0–12.0)	6.5 (5.0–11.5)	6.4 (4.7–12.0)	5.0 (3.0–12.0)	7.9 (2.9–8.3)	6.5 (3.0–12.0)	4.0 (3.0–12.0)	5.1 (2.9–12.0)
AUC_0–24_, ng/(h·mL)^b^	89 (66)	261 (137)	492 (373)	562 (374)	—	82 (71)	144 (60)	283 (115)	304 (143)	—
CL/F, L/h^b^	50 (16)	42 (25)	29 (33)	34 (17)	38 (23)	60 (55)	50 (16)	50 (23)	61 (20)	59 (30)
V_SS_/F, L^b^	2754 (348)	2597 (430)	2515 (289)	2232 (712)	2648 (471)	3368 (286)	3866 (1381)	3421 (1077)	2719 (504)	3410 (990)
t_1/2_, hours^b^	45 (27)	52 (18)	71 (52)	62 (23)	60 (33)	46 (33)	56 (19)	50 (16)	40 (10)	47 (20)

^a^ Based on observed values.

^b^ Based on population PK analysis.

AUC_0–24_, area under the plasma concentration–time curve from time 0–24 hours; CL/F, oral clearance; C_max_, maximum observed concentration; PK, pharmacokinetics; t_1/2_, apparent elimination half-life; t_max_, time to C_max_; V_SS_/F, volume of distribution; SD, standard deviation.

Assessment of the impact of patient characteristics and concomitant treatment revealed that higher weight was statistically significantly associated with increased volume of distribution (V_SS_/F; *p* < 0.01), and that increased age was significantly associated with increased CL/F (*p* < 0.01). V_SS_/F increased with 27 L by every kilogram increase in weight, whereas CL/F increased with 4.2 L/h with every year increase in age. There was no significant relationship between sex, height, ADHD diagnosis, or concomitant treatment with a stimulant and PK parameters. There was no apparent difference in plasma vortioxetine concentrations between white patients and black/other race patients. After the last dose, the median of dose-normalized (to 10 mg) C_max_ values was 11.6 ± 11.3 ng/mL among white patients and 10.5 ± 8.5 ng/mL among black/other race patients.

### CYP2D6 genotyping

A total of two patients (one child [15 mg cohort], one adolescent [5 mg cohort]) were poor metabolizers of *CYP2D6* and one child (5 mg cohort) was an ultrarapid metabolizer of *CYP2D6*. The mean vortioxetine CL/F was lower in the *CYP2D6* poor metabolizers than in the *CYP2D6* intermediate (*n* = 15) or extensive metabolizers (*n* = 30).

### Safety and tolerability

The overall rate of treatment-emergent adverse events (patient- and investigator-reported) was 79% among children and 75% among adolescents. There were no serious adverse events or adverse events leading to withdrawal during the study. The majority of adverse events were mild (∼80%), with one severe event (headache), and there was no apparent difference in the intensity of events between age or dose groups. The majority of adverse events (>60%) had an onset during the first 6 days of dosing, and more than 75% of adverse events resolved within 4 days of onset. The most common treatment-emergent adverse events (incidence ≥5% in the total population) were headache, nausea, sedation, upper abdominal pain, fatigue, vomiting, decreased appetite, and irritability (Table 3).

**Table 3.** Most Common (≥5% Overall Incidence) Treatment-Emergent Adverse Events (Self-Reported or Investigator-Identified)

*Patients,* n *(%)*	*Children (*n = *24)*	*Adolescents (*n = *24)*	*Total (*N = *48)*
Headache	5 (21)	7 (29)	12 (25)
Nausea	3 (13)	8 (33)	11 (23)
Sedation	4 (17)	7 (29)	11 (23)
Upper abdominal pain	7 (29)	1 (4)	8 (17)
Fatigue	1 (4)	5 (21)	6 (13)
Vomiting	4 (17)	2 (8)	6 (13)
Decreased appetite	1 (4)	2 (8)	3 (6)
Irritability	1 (4)	2 (8)	3 (6)

**FIG. 4.**
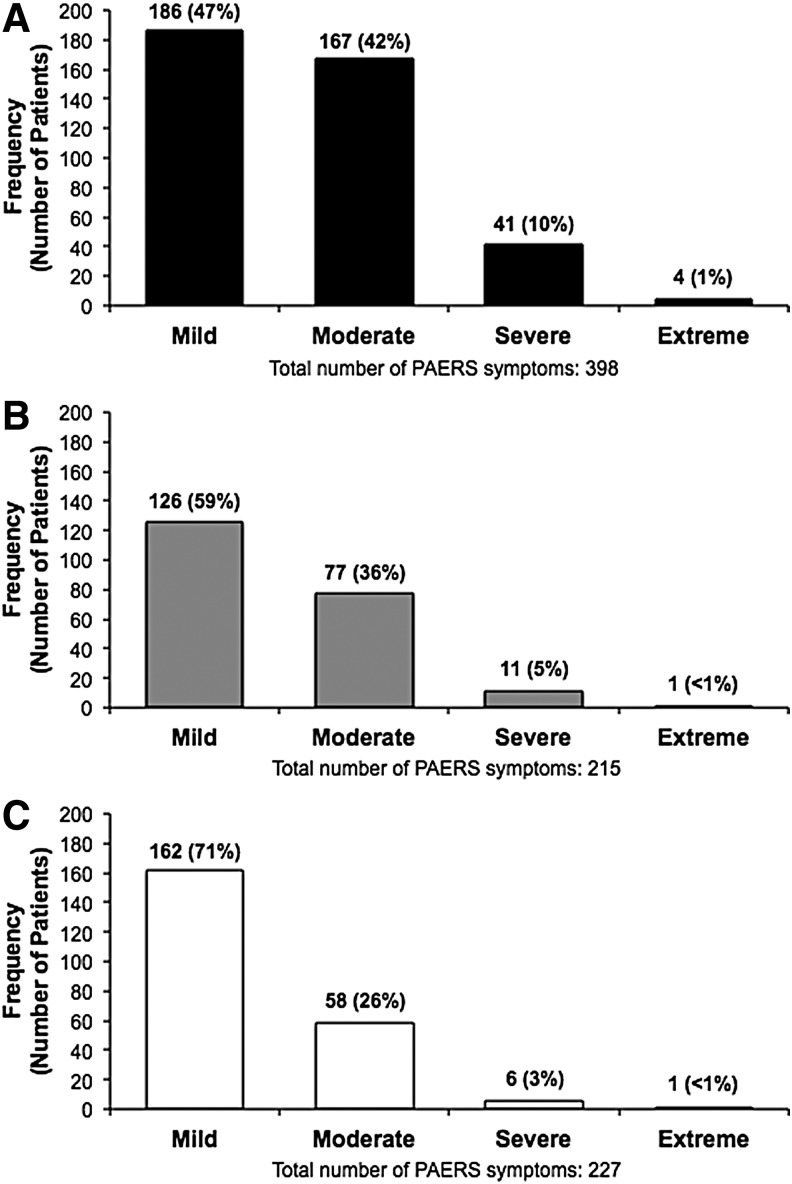
Distribution of all PAERS items for the combined cohort at baseline **(A)**, day 2 **(B)**, and day 14 **(C)**. PAERS, Pediatric Adverse Event Rating Scale.

The total number of PAERS symptoms reported by the total population on days 2 and 14 was lower than that reported at baseline, and there was a larger proportion of mild symptoms on these days than baseline reports (Fig. 4).

There were no clinically significant patterns in the mean laboratory values, vital signs, weight changes, or ECG parameter values, and there were no apparent differences in these parameters between age groups or cohorts. Based on the C-SSRS, no suicidal behavior or preparatory actions toward suicidal behavior were reported at baseline or during the study. Overall, 35% of all patients had a history of suicidal ideation. During the study, four patients (three adolescents, one child) reported suicidal ideation, three of whom had reported suicidal ideation at the baseline visit.

## Discussion

Vortioxetine, at doses of 5, 10, 15, or 20 mg q.d., was safe and well tolerated in this pediatric population (ages 7–17 years). The results suggest that exposure to vortioxetine, in terms of C_max_ and AUC_0–24_, was generally lower in adolescents than in children. The difference was seen both after the initial dose (when patients remained at the study site) and after the final dose, although the difference was greater after the final dose. Because vortioxetine is primarily metabolized through *CYP2D6*, enzyme variability also may play a role in these differences; however, a difference in *CYP2D6* genotype between adolescents and children does not appear to explain this difference in oral clearance, because the distribution of inferred metabolic status for *CYP2D6* was similar between children and adolescents.

The greater difference after the final dose may be partially related to poorer adherence among outpatient adolescents than among the younger children. Nonadherence will lead to an incorrect dose used in the PK analysis, which will subsequently result in overestimated oral clearance values; however, the formal compliance assessment in this study revealed no sign of noncompliance, neither among the children nor the adolescents. Compared with adult data (Areberg et al. 2014), exposure was similar for children but lower for adolescents, which again might indicate good adherence for the children (under supervision from parents) but poorer adherence for adolescents (possibly because of overall reduced supervision from caretakers when compared with children). Although self-reported adherence was very high, this could not be definitively confirmed, as patients were not required to remain at the study site throughout the study.

Although this study was not statistically powered for a formal drug interaction potential assessment, the results from the population PK analysis indicate that there was no significant relationship between ADHD diagnosis or between concomitant treatment with a stimulant and PK parameters. This is important given that child patients with ADHD often have comorbid depression or anxiety and there is a frequent use of stimulants in these patients (Schatz and Rostain 2006; Daviss 2008). The results also suggest that the tolerability profile is generally similar to that seen in adults, with gastrointestinal events and headache being the most common adverse events.

PK studies are useful for providing important information about how best to dose medications in children and adolescents (Moreno et al. 2007). The development of evidence-based dosing strategies is particularly important for antidepressants because clinical trials often have a high placebo response rate, making it difficult to demonstrate efficacy and because antidepressants can be associated with serious adverse events in pediatric patients (Findling et al. 2006). Historically, many of the dosing strategies in pediatric clinical trials assessing antidepressants for the treatment of MDD were not supported by PK data (Findling et al. 2006). Thus, dosing of these medications in clinical trials and general practice is usually adjusted based on body weight. This adjustment of the dose may result in subtherapeutic doses yielding negative results or in too high dosing resulting in a poor safety signal (Moreno et al. 2007).

To respond to regulatory requests (in the European Union and the United States), this initial international pediatric PK study was designed to determine the PK of vortioxetine in children and adolescents. The PK results, along with the safety and tolerability data, from this study provide support for the use of the doses of vortioxetine tested (5–20 mg q.d.) and the uptitration scheme employed in pediatric efficacy and safety studies with vortioxetine. A strength of the study is that the criteria provided a patient population that is representative of that seen in clinical practice (i.e., “ecological validity”) because it included those with the full spectrum of diagnoses typically seen in the real world, including patients with concurrent ADHD, mixed depression, or anxiety disorder (according to DSM-IV-TR), and those who had prior/current exposure to stimulants. The study also represented a broad age range of children and adolescents (i.e., 7–17 years) and included youths of both genders and from more than one race.

The information obtained from this PK study will facilitate the implementation of future clinical efficacy studies of vortioxetine in children and adolescents. In addition, an open-label extension phase to this trial has been conducted and the results will be reported in a separate publication. Results from this extension phase will provide valuable experience with the long-term use of vortioxetine in children and adolescents.

### Limitations

Limitations to this study include the lack of a placebo arm, relatively small sample size, and short duration. This precludes making any conclusions regarding the overall efficacy and safety of vortioxetine in this pediatric population. Another limitation was that adherence to pharmacotherapy was not definitively confirmed because patients were not required to remain at the study site for the entire duration of the study. Finally, although the heterogeneity in the patient population is largely a strength (in that it reflects the real-world circumstances), heterogeneity has the potential to confound results.

## Conclusion

This trial suggests that at the doses studied, acute treatment with vortioxetine is generally well tolerated in pediatric patients, and that the PK profile of the drug supports the use of doses evaluated (5–20 mg q.d.) and the uptitration schedule used in future pediatric efficacy and safety studies.

## Clinical Significance

Results from this study suggest that the doses of vortioxetine tested (5–20 mg q.d.) and the uptitration scheme employed appear to be appropriate for use in clinical trials that will more definitively evaluate the efficacy and safety of vortioxetine in pediatric patients.
